# Supplementary material for: EEG in the classroom: Synchronised neural recordings during video presentation
Source: Sci Rep. 2017 Mar 7;7:43916. doi: 10.1038/srep43916 (PMC5339684; doi:10.1038/srep43916)
Supplement: Supplementary Materials [file srep43916-s1.pdf]

Supplementary Material - EEG in the classroom: Synchronised neural recordings during video presentation

Andreas Trier Poulsen[1,+,*], Simon Kamronn[1,+], Jacek Dmochowski[2,3], Lucas C. Parra[3], and Lars Kai Hansen[1]

[1]Technical University of Denmark, DTU Compute, Kgs. Lyngby, Denmark
[2]Stanford University, Department of Psychology, Palo Alto, USA
[3]City College of New York, Department of Biomedical Engineering, New York, USA
[*]atpo@dtu.dk
[+]these authors contributed equally to this work

Contents

S1 Supplementary Tables and Figures **2**

S2 Experimental setup **3**
S2.1 Stimulus . . . 3
S2.2 Individual viewings . . . 4
S2.3 Joint viewing . . . 5
S2.4 Questionnaires and general information about the subjects . . . 5

S3 Hardware **5**
S3.1 Emocap . . . 5
S3.2 Tablet . . . 6
S3.3 Synchronisation . . . 6
S3.3.1 Individual viewing . . . 6
S3.3.2 Joint viewing . . . 7

S4 Questionnaires **7**

References **11**

S1 Supplementary Tables and Figures

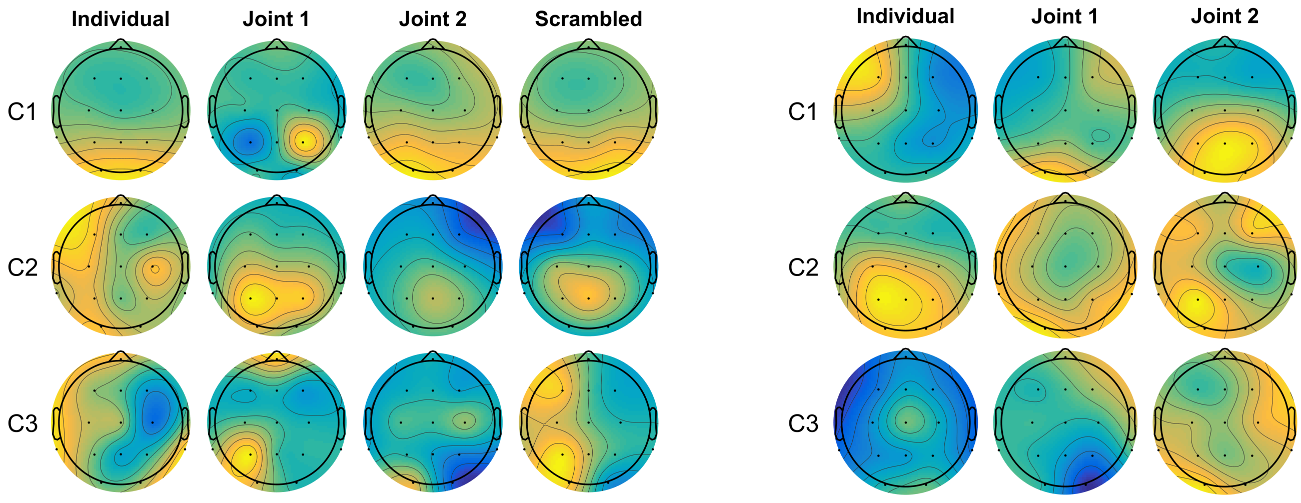

(a) *Sophie's Choice* **(b)** *Baseline video*

Figure S1. The corresponding scalp projections of the first three components obtained from the correlated component analysis (CorrCA) of each of the subject groups watching *Sophie's Choice* and a baseline video for the first time. For each component, CorrCA finds one shared set of weights for all subjects in the group. Four distinct groups of subjects watched videos in different scenarios: individually on a tablet computer (*Individual*), individually with order of scenes scrambled in time (*Scrambled*), jointly in a classroom as seen in Fig. 1 (*Joint 1* and *Joint 2*). For each projection, the polarity was normalized so the value at Cz is positive. Note that the *scrambled* group did not watch the Baseline video.

Table S1. Correlation coefficients between the results obtained in a laboratory setting[1] and those obtained in the present study (groups *Individual, Joint 1* and *Joint 2*). Inter-subject correlation (ISC) measures similarity of responses between subjects for first and second viewings (v1,v2) and the inter-viewing correlation (IVC) measures similarity within-subject between the two views. Values are calculated using the second correlated component recorded while watching *Bang! You're dead.* **: $p < 0.01$, *: $p < 0.05$.

	ISC v1	ISC v2	IVC
Individual	0.05	0.08	0.10*
Joint group 1	0.20**	0.12*	0.28**
Joint group 2	0.27**	0.00	0.10*

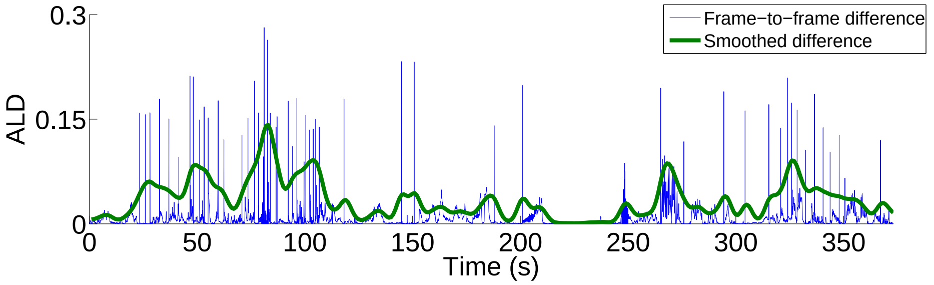

Figure S2. Comparison between the frame-to-frame average luminance difference calculated from *Bang! You're Dead* and the one which have been smoothed with kernel smoothing. Each large value corresponds to a change in camera position.

Table S2. Scenes described as having the strongest impression by subjects. Based on the 30 subjects which saw *Sophie's Choice* with unscrambled narrative. Subjects were asked, in a post-experiment questionnaire, to describe the scenes that made the strongest impression on them. Their answers have here been collected in the nine groups. The subjects mentioned 1.67 scenes on average (0.76 std.). *: Note: only the girl is taken away from her mother, however 47 % of the subjects indicated that both children were taken.

Scene	Number of times mentioned (%)
When both children are taken*	14 (47 %)
The mother is forced to choose between her children	11 (37 %)
The girl is taken from her mother	8 (27 %)
When the girl is screaming at the end	8 (27 %)
When the mother cries at the end	3 (10 %)
Panorama of Jews in lines	2 (7 %)
When the Nazi officer speaks to the mother	2 (7 %)
When I recognised Meryl Streep	1 (3 %)

S2 Experimental setup

To avoid gender having a factor in the results 42 female subjects were recruited with an average age of 22.4 years, distributed with minimum, median, and maximum ages of 18, 22, and 32 respectively. All subjects signed a consent for the use of data, video and image.

The subjects were divided into two groups, with one group of 24 subjects watching the films alone (*Individual viewing*) and another group of 18 subjects subdivided into two groups of nine, which watched the films together (*Joint viewing*). There were taken precautions to ensure that the subjects participating in the same joint viewing, did not know each other beforehand to avoid unwanted confounding factors. The group with individual viewings were additionally evenly divided into a group watching the films with the order of the scenes scrambled and a group watching the film clips normally.

S2.1 Stimulus

One of the goals of the experiments presented here was to recreate the results in ([1]), where the subjects were shown clips from three different films; *Bang! You're Dead* (1961) directed by Alfred Hitchcock, *The Good, the Bad, and the Ugly* (1966), a western directed by Sergio Leone, and a control film of a natural outdoor scene on a college campus. The Hitchcock film produced great results and the same clip was therefore included in the experiments presented here. The western, however, did not produce as many significant times of correlation, and it was decided to replace this clip with one from *Sophie's Choice* (1982) directed by Alan J. Pakula.

The clip from *Sophie's Choice* depicts a young Polish mother on her way to concentration camp during World War II, with her two children. She is accosted by a German officer, who forces her to choose which of her children lives or dies. The dialogue in the film clip is in German. The same film clip was used by ([2]), where the subjects were investigated for emotion-related changes using fMRI and viewer feedback rating. The study found a monotonic increasing response with the

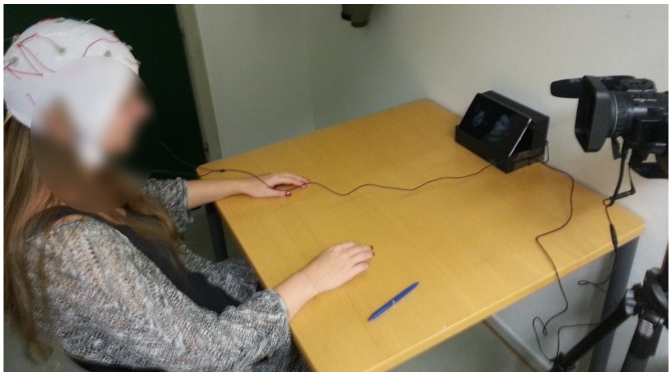

Figure S3. Experimental setup for single viewings.

highest scoring emotions being ”horror”, ”hate”, ”fear”, and ”anger”.

To act as a control, a video was recorded of the escalators Kgs. Nytorv metro station in Copenhagen. This setting was chosen to eliminate the argument that the joint engagement is found for vision of a body versus non-body stimulus. The metro station was chosen as it was rationalised that the passengers getting on the metro in this station, were in less of a hurry compared to other stations, thereby reducing any excitement of people running to catch their train.

Each clip had a length of approximately 6 minutes and were shown twice to each subject. For each viewing the order was randomised, but the same order was used the second time the clips were shown. A combined video was created for each of the six possible permutations of the order of the clips, starting with a 10 second 43 Hz tone for use in post processing synchronisation, and 20 seconds black screen between each film clip. At the end of the video the subject was presented with a text announcing that the video was over, to avoid the subject wondering if they just saw the last clip, between each clip. The total length of the video amounted to 39 minutes.

In ([1]) the order of the scenes in *Bang! You're Dead* were scrambled to investigate the response when the meaning of the film was lost. The same approach was used in this thesis for both *Bang! You're Dead* and *Sophie's Choice*. Since the control video was intended not to carry any meaning, this was left out of the video with scrambled scenes resulting in only two permutations and a length of 23 minutes.

S2.2 Individual viewings

24 subjects were used for the individual viewings, which were conducted in a small office as seen on figure S3. The film was shown on a Google Nexus 7 tablet, with a 7” (17.8 cm) screen with the subject hearing the films through in-ear headphones to avoid wires crossing the head. The headphones had a noise dampening effect which was important due to some of the recordings being made in office hours. Subjects was instructed to sit straight, and avoid movements which could cause artefacts in the EEG, such as chewing, heavy breathing, and limb movement. They were also instructed to look within the frame of the screen to reduce eye artefacts, but was also told to relax and watch the film.

Before the viewing started, each subject drew without replacement for whether the films should be scrambled or not, and afterwards used a dice to decide the order of the film clips.

The subjects were filmed with a camera receiving sound input directly from the tablet as well as from an external microphone. An electric spark was used for post processing synchronisation between the spark showing in the EEG and its clicking sound on the camera recording. As the camera also recorded the sound output from the tablets, the time interval between the spark and the time of the 43 Hz tune could be calculated, and from this the time of start for each film clip.

The lighting in the room was controlled by blacking out the office window and only having an architect lamp on, to ensure the subjects were visible on the camera in the dim light.

S2.3 Joint viewing

The joint viewing experiment is an expansion of the single viewing experiment presented by ([1]). Since recording EEG from nine subjects simultaneously is relatively new territory and presents new obstacles, the experimental setup deviates from the one in the single viewing in some areas.

Different approaches to the placement of the subjects in the room in relation to the screen and to each other were considered. It was decided to go for a ”cinema experience”, with all nine subjects sitting on a line of chairs. By instructing the subjects to keep their eyes within the screen, as in the single viewings, they were not able to directly see the facial expressions of one another. As the films were watched on a projector it was possible to both regulate the distance from the subject to the screen and the length of the diagonal of the picture projected on the screen. It was decided to keep the viewing angle from one corner of the screen to the opposite corner similar to the one in the single viewings. By assuming the line of sight was orthogonal to the screen the relation

$$\text{angle} = \tan^{-1} \frac{\text{screen diagonal}}{\text{distance to screen}} \tag{1}$$

was used to find the maximal angle the eye could move while still viewing the screen. In the single viewings the distance from head to screen varied from 70-90 cm, giving angles of maximal eye movement of 11.2° to 14.3°. The distance from the subject in the centre chair to the screen was measured to be 450 cm and 490 cm for the outermost placed subjects. With a screen diagonal of 102 cm this resulted in angles of maximal eye movement between 11.8° and 12.8°.

The recordings were done in a larger room, to accommodate all the subjects, and the sound from the films was played through loudspeakers, to avoid the emotional distance which noise dampening headphones might produce. On the basis of creating similar lighting conditions as in the single viewing experiment the windows were blacked out and four lamps placed strategically to avoid shining a light in the eyes of the subjects, but still illuminating them for the purpose of filming them. For the joint viewings the subjects were filmed using a GoPro Hero 2. The image and sound quality of the GoPro was not as good as the original camera (especially in the dim lightening), but it had the benefit of being unobtrusive and could be placed directly in front of the subjects. The recording tablets were placed on tables directly behind the subjects to avoid loss of connection from transmitters with poor transmitting distance.

S2.4 Questionnaires and general information about the subjects

Before the EEG recordings all subjects were asked to fill out a questionnaire. Apart from asking relevant physiological questions, it was also chosen to ask the subjects to evaluate their level of proficiency in German, because of the German dialogue in *Sophie's Choice*.

After viewing the films the subjects were asked to answer another questionnaire regarding whether they knew the scenes beforehand and which scenes had the biggest impact on them. Subjects viewing the films with scrambled scenes were also asked to describe the plot in the two films. This was both done to evaluate and possibly subdivide the subjects based on their understanding as well as for a comparison with the results gained from the EEG.

S3 Hardware

Research grade EEG equipment is often very expensive, time-consuming to equip, and immobile. Using smaller consumer grade hardware has thus many advantages if it is able to measure the required signals adequately. Part of the motivation for the experiment presented in this article was to validate if the hardware used is sufficient for the paradigm, in what areas it may be advantageous, and in what areas it is lacking.

S3.1 Emocap

To conduct the experiments the mobile 14 channel consumer EEG headset Emotiv EPOC was rebuild to a wireless cap based on EasyCap, the Emocap. The sampling frequency of the ADC is 2048Hz but since the EPOC only have one ADC the data is

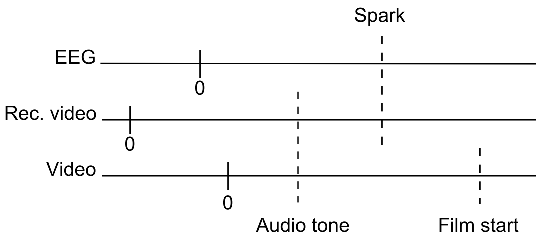

(a) Individual viewing.

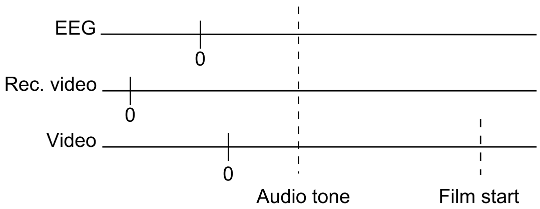

(b) Joint viewing.

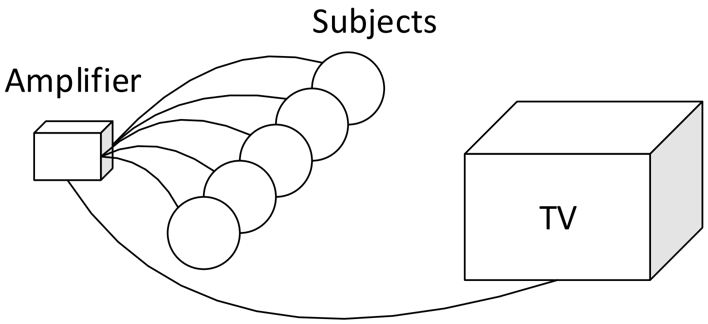

(c) Joint viewing setup.

Figure S4. Illustration of the synchronisation paradigms for the individual and joint viewing experiments.

sampled sequentially which means that the effective sample frequency of each channel is 128Hz (including Common Mode Reference and Driven-Right-Leg electrodes).[3] Each sample is assigned a number from 0 - 128 in the EPOC in order to ensure detection of packet loss on a sub-second time-scale. The EPOC has previously been validated against the Biosemi Active-II device with 64 channels using an imagined finger tapping paradigm.[4]

The electrode placement of the Emocap follows the 10-20 system in naming and placement, but with only 14 measurement electrodes the configuration is specific to this setup.

S3.2 Tablet

To acquire and record data from the Emocap it is possible to use a computer or a mobile device supporting direct access to the USB port. In this experiment tablets of the model Asus Nexus 7 were used. The processing power of the device is much greater than needed for this application and the tablet has previously been shown to work well with the EPOC.[4]

S3.3 Synchronisation

Experiments involving a stimulus are highly dependent on temporal alignment if the objective is to compare the results across modalities or recordings. To synchronise EEG recordings with the film, two methods were employed based on the experimental condition.

S3.3.1 Individual viewing

For the subjects viewing the films alone the method of synchronisation was based on the theory that the electro magnetic wave, generated by creating a powerful spark, would induce a small current in the wires from the electrodes. This was confirmed using a piezoelectric spark generator normally used to ignite a Bunsen burner. Based on the length of the spark it was estimated that the spark was around 2 kV and very low amperage. When used approximately 2 centimetres from the electrodes a spike with much higher amplitude than the surrounding artefact free EEG was observed. Generation of the spark also emitted a noise that was distinguishable in the audio track of the recorded video. This method was hence used to synchronise the EEG with the

recorded video in single subject experiments. To synchronise the recorded video with the film showed on the tablet, the audio output from the tablet was connected to the input of the camera in parallel with a microphone. At a fixed time before the first film clip a 43Hz sine wave was played to make this part of the synchronisation easier, as illustrated in figure S4a.

S3.3.2 Joint viewing

In the experiments involving simultaneous EEG recordings of multiple subjects, a method of inducing a current simultaneously in all EEG recordings was necessary. Though experiments with creating more powerful sparks with estimated voltages of up to 60 kV, the field from the circuit was not visible in the EEG, meaning that either the signal strength was not adequate or the DRL circuit in the Emocap managed to suppress the signal.

S4 Questionnaires

Table S3. Information regarding the subjects, under which condition they saw the movies, and how they perceived them. Self-reported German proficiency rates is scored from 1 to 4, with 1 being equal to "None". **S** = Scrambled scenes, **NS** = Non-scrambled scenes, **J1** = The first joint viewing, **J2** = The second joint viewing.

Subject no.	Condition	Order of movies	Age	Hours of sleep	German proficiency	Right handed	Seen the movies before	Understood the movies
16	S	1	20	7	3	Yes	No	Yes
10	NS	2	21	9	3	No	No	Yes
9	S	1	19	9	2	Yes	No	Yes
15	S	1	22	6	2	Yes	No	Yes
3	NS	2	20	7,5	2	Yes	No	Yes
6	NS	1	20	7,5	3	Yes	No	Yes
11	S	1	18	10	2	Yes	No	Yes
2	NS	5	20	9	3	Yes	No	Yes
4	NS	6	21	7	2	Yes	No	Yes
5	NS	1	21	9	2	Yes	No	Yes
14	S	1	20	9	2	Yes	No	Yes
12	S	1	21	8,5	2	No	No	Yes
13	S	2	20	8,5	1	Yes	No	Yes
7	NS	3	21	7	2	Yes	No	Yes
1	NS	2	19	8	1	Yes	No	Not Sophie's
8	S	2	19	8	1	Yes	No	Not Bang!
23	NS	4	24	7	2	Yes	No	Yes
22	S	1	25	8	2	Yes	No	Not Bang!
24	NS	3	25	8	2	Yes	No	Yes
21	S	1	25	5	2	Yes	No	Yes
17	S	1	21	7	1	Yes	No	Yes
19	S	1	21	7	2	No	No	Yes
18	NS	6	22	7,5	1	No	No	Yes
20	NS	6	23	8	2	Yes	No	Yes
33	J1	6	32	7	3	Yes	No	Yes
28	J1	6	25	8	2	Yes	No	Yes
30	J1	6	24	8	2	Yes	No	Yes
31	J1	6	26	8	1	Yes	No	Yes
27	J1	6	25	5,5	2	Yes	No	Yes
25	J1	6	21	8	2	Yes	No	Yes
29	J1	6	25	10	2	Yes	No	Maybe
26	J1	6	21	8	1	Yes	No	Yes
32	J1	6	25	7	2	Yes	No	Yes
34	J2	6	22	10	2	Yes	No	Yes
36	J2	6	20	9	1	Yes	Sophie's	Yes
35	J2	6	25	8,5	2	Yes	No	Yes
41	J2	6	25	8	2	Yes	No	Yes
37	J2	6	25	9	2	Yes	No	Yes
42	J2	6	22	6	3	Yes	No	Yes
40	J2	6	22	6,5	3	No	No	Yes
39	J2	6	24	8	2	Yes	Sophie's	Yes
38	J2	6	23	8	1	Yes	No	Yes

Questionnaire before EEG-measurement

**Name:**		**subject no.:**	
**Movie:**			

1. Are you right handed? Yes ☐ No ☐
2. Normal sight/corrected to normal vision Yes ☐ No ☐
3. Normal hearing: Yes ☐ No ☐
4. How many hours have you slept last night? ____________
5. Age ____________
6. Do you have a psychiatric record? Yes ☐ No ☐
7. Do you have a neurologic record? Yes ☐ No ☐
8. Have you ingested drugs or medication the last 24 hours? Yes ☐ No ☐
   a. If yes, which? ____________
9. Level of German proficiency
   a. Fluent ☐
   b. Good understanding of the language ☐
   c. Basic understanding of the language ☐
   d. None ☐
10. Are you interested in participating in future experiments? If you answer ”Yes”, your sex, age and contact information will be saved for future use. Yes ☐ No ☐
11. Can we use pictures of the experimental setup, where you appear for our Master thesis, article or other things that regard this experiment? Yes ☐ No ☐

Mobiles and other electric equipment have to be removed before the experiment.

I hearby confirm, that I agree to participate in a experiment with EEG recordings during viewing films. I am informed that I participate voluntarily and that I can, at any time and without reasons, can redraw my consent to participate.

Date : ________________ Signature : ____

Questionnaire after EEG-measurement

**Name:**		**Subject no.:**	

1. Had you seen the movies before
   a. The black/white movie — Yes ☐ No ☐
   b. The movie in colour — Yes ☐ No ☐
2. Did you understand the movie in german? (the one in colour) — Yes ☐ No ☐

3. Which scenes made the strongest impression in the black/white movie?

____

____

____

____

4. Which scenes made the strongest impression in the movie in colour?

____

____

____

____

References

1. Dmochowski, J. P., Sajda, P., Dias, J. & Parra, L. C. Correlated components of ongoing EEG point to emotionally laden attention - a possible marker of engagement? *Frontiers in human neuroscience* **6**, 112 (2012).

2. Raz, G. *et al.* Portraying emotions at their unfolding: a multilayered approach for probing dynamics of neural networks. *NeuroImage* **60**, 1448–61 (2012).

3. Emotiv. EPOC specifications (2012).

4. Stopczynski, A., Stahlhut, C., Larsen, J. E., Petersen, M. K. & Hansen, L. K. The Smartphone Brain Scanner: A Portable Real-Time Neuroimaging System. *PloS one* **9**, e86733 (2014).
